# Developmental and activity-dependent plasticity of filiform hair receptors in the locust

**DOI:** 10.3389/fphys.2013.00070

**Published:** 2013-08-23

**Authors:** Hans-Joachim Pflüger, Harald Wolf

**Affiliations:** ^1^Department of Neurobiology, Institute of Biology, Fachbereich Biologie, Chemie, Pharmazie, Freie Universität BerlinBerlin, Germany; ^2^Wallenberg Research Centre, Stellenbosch Institute for Advanced Study, Stellenbosch UniversityStellenbosch, South Africa

**Keywords:** insect flight, filiform hair receptors, wind receptors, developmental plasticity, interneuron

## Abstract

A group of wind sensitive filiform hair receptors on the locust thorax and head makes contact onto a pair of identified interneuron, A4I1. The hair receptors' central nervous projections exhibit pronounced structural dynamics during nymphal development, for example, by gradually eliminating their ipsilateral dendritic field while maintaining the contralateral one. These changes are dependent not only on hormones controlling development but on neuronal activity as well. The hair-to-interneuron system has remarkably high gain (close to 1) and makes contact to flight steering muscles. During stationary flight in front of a wind tunnel, interneuron A4I1 is active in the wing beat rhythm, and in addition it responds strongly to stimulation of sensory hairs in its receptive field. A role of the hair-to-interneuron in flight steering is thus suggested. This system appears suitable for further study of developmental and activity-dependent plasticity in a sensorimotor context with known connectivity patterns.

## Introduction

To serve the requirements of behavior in different life stages and different biological habitats, the nervous system must exhibit a considerable degree of flexibility, particularly in holometabolous insects. In the tobacco hawkmoth, *Mandua sexta*, for example, larva and adult exhibit different life history traits associated with their respective functions, occupy different ecological niches and show different behaviors. The predominant larval behaviors are crawling, feeding, defensive behaviors, and moulting, whereas in adults these are walking and flying, feeding, and all behaviors associated with courtship and reproduction. These changes in the nervous system are induced by and dependent on developmental hormones including ecdyson and juvenile hormone, and they occur predominantly at pupation and during metamorphosis (Riddiford et al., 2003). For motor neurons that persist from larva to adult and innervate muscles that have very different contractile properties in these two life stages, it has been well established that dendritic morphologies and electrical properties change markedly during development (Kent and Levine, 1988; Levine and Weeks, 1990; Duch and Levine, 2000; Tissot and Stocker, 2000; Weeks, 2003). Not only hormones but also neuronal activity has a role in this developmental plasticity (Duch and Mentel, 2003).

Such changes are not restricted to the motor system. Some persisting sensory receptors also exhibit structural changes with respect to their axonal arbors (Levine et al., 1985; Kent and Levine, 1988; Kent et al., 1996; Tissot and Stocker, 2000). In general, these changes follow similar patterns as observed in motor neuron dendrites: retraction of larval axonal branches is followed by a more elaborate outgrowth to generate the adult axonal arbors. However, the changes in the sensory axonal arbors are less conspicuous than those in the motor neuron dendrites. Corresponding changes are observed in the relevant sensory-motor circuitry (Gray and Weeks, 2003).

In hemimetabolous insects such as locusts, corresponding developmental changes are less obvious. The first nymph already seems like a miniature version of the final adult animal, except for the missing wings that develop postembryonically. In these insects most structural changes during development would thus appear to be associated with wing development and with the subsequent commencement of flight behavior (e.g., Altmann et al., 1978). Nonetheless, as the insect grows and expands its body surface, sensory cells are added virtually everywhere, particularly mechanosensory hairs. This has been studied probably in most detail in the cerci of crickets (Murphey, 1986; Dangles et al., 2006, 2008; Mulder-Rosi et al., 2010; Miller et al., 2011), with regard to their endowment with wind sensitive hairs. Gradual changes in the strength and localization of synaptic contacts are essential here to accommodate the increasing number of sensory cells impinging on a given central nervous interneuron. These changes appear relatively small, however, compared to the complete retraction and new outgrowth of whole neuronal arbors during metamorphosis.

Here, we present a model system in the locust that allows study of developmental plasticity in sensory projections and connectivity. Wind sensitive hairs on the head and especially the thorax make monosynaptic connections to an identified interneuron, A4I1. These sensory hair-to-interneuron connections changes during nymphal development, and these changes depend on neuronal activity with regard to both morphology and synaptic contact. We further present a first experiment addressing possible functions of this sensory hair-to-interneuron system in locust flight control.

## Results and discussion

### The filiform hair system of the locust prothorax and head

Filiform hairs are extremely sensitive to wind—air current—or to local movement of air particles—low-pitched sound and infrasound—such as occur in the near field of a loudspeaker. Filiform hairs are known from the cerci of many insects, such as cockroaches and crickets (Murphey, 1986; Dangles et al., 2006, 2008; Mulder-Rosi et al., 2010; Miller et al., 2011), and from caterpillars, where they mediate escape responses (Tautz and Markl, 1978; Gnatzy and Tautz, 1980; Blagburn and Beadle, 1982; Pflüger and Tautz, 1982; Bacon and Murphey, 1984; Ogawa et al., 2006; Heys et al., 2012). Spiders possess similar, highly sensitive hair sensilla known as trichobothria. They are also involved in escape responses (Gronenberg, 1989) and in prey capture as well (Barth et al., 1995).

These filiform hairs enable insects and spiders to detect wind from the wing beat of predatory wasps or even the wind puff produced by the protruding tongue of a striking toad (cited in Camhi, 1984). The individual filiform hair exhibits clear directional sensitivity (Dagan and Volman, 1982). Due to the spatial arrangement of hairs with different directional preferences on the cercus of a cricket or cockroach, stimuli from all directions are detected by the ordered array of receptors, and stimulus direction is coded accordingly (Murphey, 1986; Dangles et al., 2006, 2008; Mulder-Rosi et al., 2010; Miller et al., 2011). The synaptic connections between these hairs and first-order interneurons are remodeled during postembryonic development (Chiba et al., 1988), although in a more gradual fashion than during metamorphosis in holometabolous insects. Such remodeling in hemimetabolous insects may nonetheless be profound.

**Figure 1:**
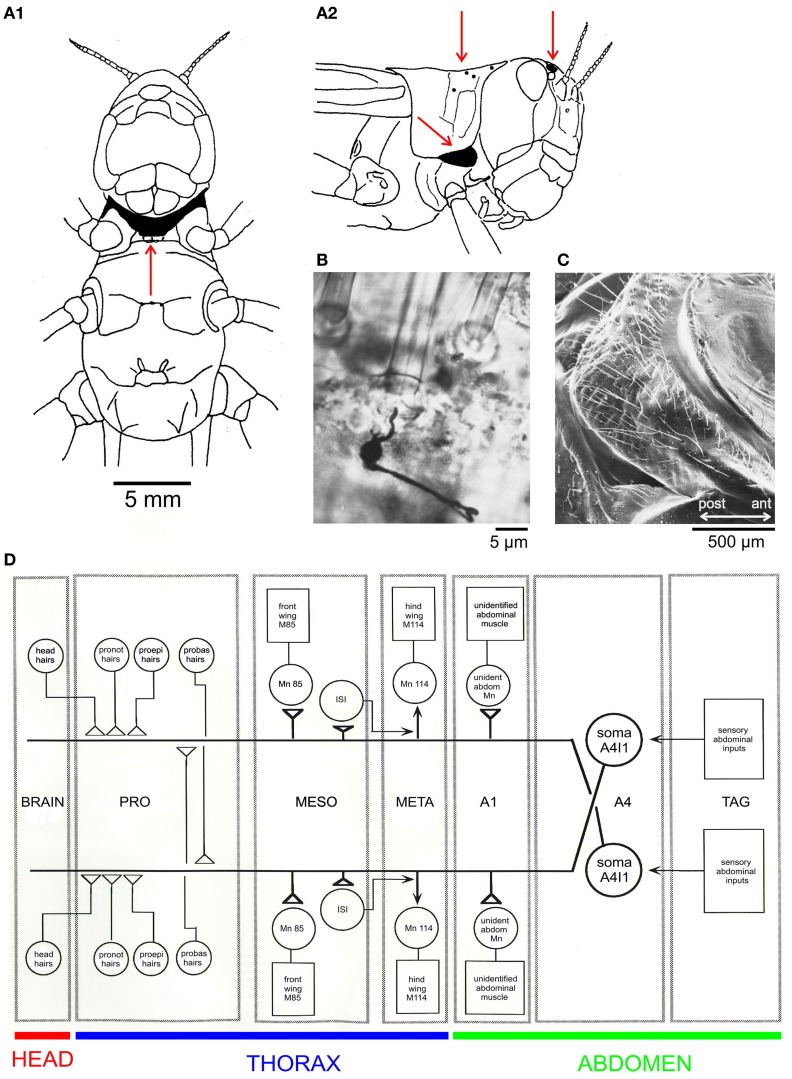
**The locust filiform hair-to-interneuron system. (A)** Schematic drawings of a locust viewed from the ventral **(A1)** and lateral **(A2)** sides; red arrows indicate locations of filiform hairs in the areas shaded in black: the ventral probasisternum **(A1)**, the lateral proepisternum (**A2**, ventral), the dorsal pronotum (**A2**, dorsal), and field 1 of the wind sensitive head hairs. **(B)** Silver-intensified cobalt fill of the peripheral sensory nerve revealing cell body and initial axon segment of a mechanoreceptive sensory neuron and its dendrite attached to the base of a filiform probasisternal hair in a whole-mount preparation (Watson and Pflüger, 1984). **(C)** A scanning electron micrograph of an adult locust probasisternum showing the array of filiform hair receptors in ventral view. **(D)** A schematic drawing of the filiform hair-to-interneuron system in the locust (Pflüger et al., 1994). Abbreviations: A1, A4, first and fourth abdominal neuromeres; ant, anterior; ISI, intersegmental interneuron; M, muscle; MESO, META, meso- and meta-thoracic ganglia; Mn, Motor neuron; probas, probasisternal; proepi, proepisternal; pronot, pronotal; TAG, terminal abdominal ganglion.

Less well known than cercal hairs are similar wind sensitive filiform sensilla on other body parts. In locusts, these occur on the frontal head and on the thorax, namely, on the ventral probasisternum, the lateral proepisternum, and the dorsal pronotum. In the first nymphal instar, there are 8 hairs on each half of the probasisternum, 2 on each proepisternum, and 3 or 4 on each half of the pronotum (Figure 1A, red arrows point to the hair receptors). During each moult new hair receptors are added, resulting in a total number of about 300 probasisternal cuticular hairs in the adult (Pflüger et al., 1994). Figure 1C shows a scanning electron micrograph of the adult probasisternum with its arrangement of filiform hairs. A single mechanosensory cell with its dendrite attached to the base of the hair shaft is revealed by a silver intensified cobalt chloride fill (Watson and Pflüger, 1984) in Figure 1B.

The filiform hairs that are present in the first nymphal instar are easily recognized in adults by their relative positions, and most conspicuously by the lengths of their hair shafts which are the longest compared to all other hairs. In addition, these are the receptor cells most sensitive to wind stimuli in adults (Pflüger and Tautz, 1982). Thus, individual filiform hairs can be monitored throughout postembryonic development.

**Figure 2:**
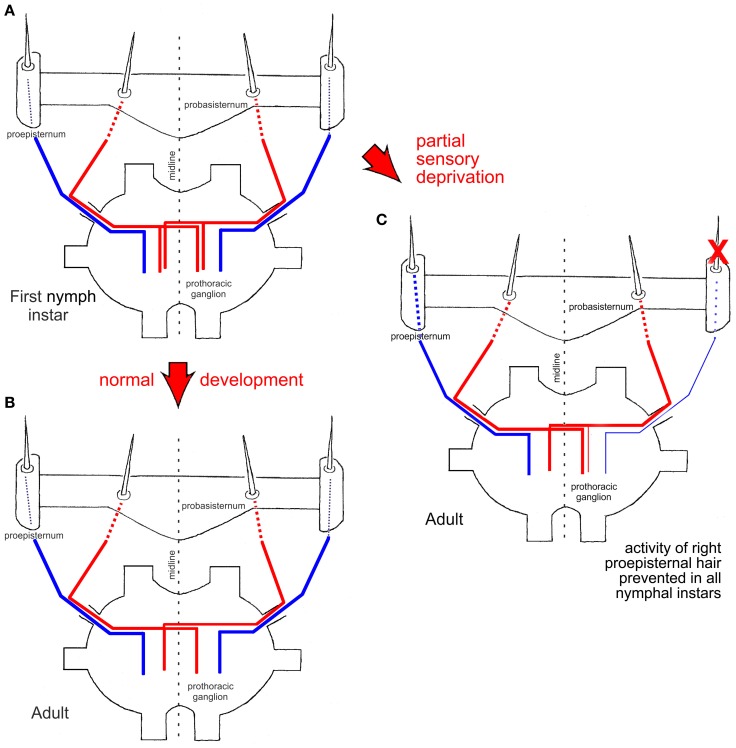
**Schematic drawings of central nervous projections of two proepisternal (blue) and two probasisternal (red) filiform hairs into the prothoracic ganglion in a first nymphal instar (A) and an adult (B) (after Pflüger et al., 1994). (C)** Experimental adult animal in which the neuronal activity of one proepisternal hair had been prevented in all nymphal instars (red X). Note that the central nervous projection of the experimentally silenced proepisternal hair was present but weaker than normal [compare to **(A)**], and also note the survival of the ipsilateral central nervous projection of an adjacent probasisternal hair. The central nervous projection of a filiform hair on the probasisternum contralateral to the manipulated side remained unaffected and exhibited the normal adult pattern. Red arrows between figure parts indicate normal and experimental situations during development, respectively.

The above mentioned hairs (Figure 1A) are part of the receptive fields of a (bilaterally symmetric) pair of projection neurons (A4I1, Figure 1D, schematic drawing; Pflüger, 1984), and all make monosynaptic connections within the prothoracic ganglion (Burrows and Pflüger, 1990; see also Figure 2). Some of the output connections of this projection neuron (A4I1) are described below.

### The central projections of filiform hairs exhibit structural dynamics in postembryonic development

The central axonal arbor of an individual filiform hair was stained by placing a blunt glass microelectrode filled with a solution of either cobalt salts or fluorescent dyes over the base of the cut hair shaft and applying currents for up to 45 min. In adult locusts (Figure 2B; see Pflüger and Burrows, 1990), the projection patterns of probasisternal hairs exhibit exclusively contralateral projections (Figure 2, red) whereas both the proepisternal and pronotal receptors have only ipsilateral projections (Figure 2, blue). In the first nymphal instar (Figure 2A; Pflüger et al., 1994), by contrast, the axonal arbors of the same probasisternal filiform hairs show both ipsi- and contra-lateral projections (Figure 2A, red), whereas those of proepisternal and pronotal hairs only reveal ipsilateral projections, like in the adult. When individual probasisternal hairs were stained in the different nymphal instars, those that were at the most lateral position of the probasisternum lost their ipsilateral axonal branch first whereas those at the most median position lost their ipsilateral branch latest, i.e., only in the final nymphal instar before the imaginal moult. Thus, there is a temporal gradient of loss of the ipsilateral branch in the projection pattern that parallels the position of the hair on the probasisternum from lateral to median. There is an increasing loss of second and higher order branches in the ipsilateral axonal arborization, and at the same time complexity of branching on the contralateral side increases in the course of consecutive nymphal instars. In contrast, proepisternal and pronotal hairs exhibit ipsilateral projections throughout all nymphal instars, and appear to undergo only synaptic refinement and pruning within the general layout of this ipsilateral branch (Pflüger et al., 1994).

In order to study the contribution of activity-dependent processes to this developmental plasticity, the activity of a proepisternal filiform hair receptor was blocked in all postembryonic stages—nymphal instars—by either immobilizing the hair shaft by wax or by cutting it close to its base immediately after each nymphal moult (Figure 2C, red X). These experimental procedures interfered only with the neuronal activity generated by the mechanoreceptor associated with the respective hair but not with the position of the hair on the cuticle. Subsequently, in adult locusts, the projection patterns of the manipulated hair were examined, as well as those of the adjacent untreated probasisternal filiform hairs, and those of the contralateral probasisternum were used as controls. Compared to normal development, the manipulated hair exhibited sparser arborizations. Most notably however was the fact that the filiform probasisternal hairs adjacent to the manipulated proepisternal hair retained their ipsilateral branches (Figure 2C). The controls on the untreated body side exhibited the normal elimination of ipsilateral arborizations, by contrast. Thus, an activity-dependent competition process obviously exists between the proepisternal and probasisternal hair receptors at least, in addition to the developmental hormonal process that shapes the final projections patterns of the mechanoreceptive cells of this sensory system (Pflüger et al., 1994).

### The A4I1-neuron: input and output connections

**Figure 3:**
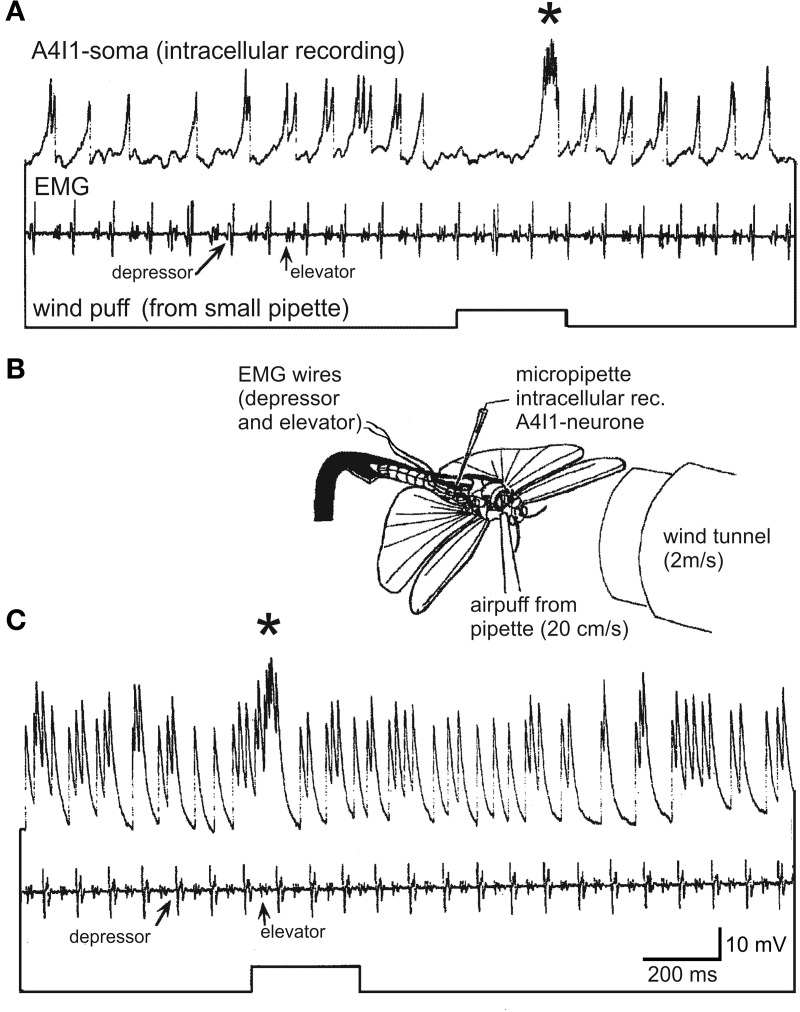
**Intracellular recording from the soma of an A4I1-interneuron (top trace), electromyogram from wing muscles (depressor and elevator, second trace), and wind puff monitor (bottom trace).** The wind puff was applied to the side of the animal where the recorded A4I1 had its axon (i.e., ipsilateral to the axon, and thus contralateral to the soma). **(B)** Experimental situation (details in text). The locust was fixed to a holder and flying upside-down, and a small window was cut into the abdomen to expose the fourth abdominal ganglion which was immobilized on a small steel platform to avoid movement. 50 μm steel wires insulated except for cut end were used for electromyograms and placed into respective muscles. The locust was flying spontaneously and without head wind from the wind tunnel in **(A)**; the wind tunnel was switched on in **(C)**.

All the above mentioned wind receptors connect directly to a first-order interneuron termed A4I1 (the term signifies that the soma is located within the first unfused, that is, the fourth abdominal ganglion). This is a projection interneuron originating in the fourth abdominal ganglion with its axon ascending contralateral to the soma and terminating within the dorsal deutocerebrum. The main input, and thus the main spike initiating zone, of A4I1 is located in the prothoracic ganglion, where all the hair receptors make their direct connections. Even the small number of wind sensitive head hairs in field 1 (>5; Figure 1A2) project to the prothoracic ganglion and make direct connections to the A4I1 interneuron there. Again, these hairs are the first in field 1 to exist in a first nymphal instar. This morphological peculiarity of interneuron A4I1 is reflected in its firing properties: An identical burst of spikes is simultaneously sent anteriorly to the brain and posteriorly toward the fourth abdominal ganglion, thus representing a perfect corollary discharge. Corresponding to this morphology, intracellular recordings from the soma show passively invading action potentials (see Figure 3) generated more anteriorly within the prothoracic spike initiation zone.

It was an intriguing result of these connectivity studies that the synaptic strengths of the filiform hair-to-interneuron connections were large indeed. Many of the individually identifiable filiform hairs exhibited gains of 1, or close to 1 (Pflüger and Burrows, 1990). That is, almost every spike in a hair receptor elicited a spike in interneuron A4I1.

The intriguing receptive field and high input gain of interneuron A4I1 beg the question of what the output connections of this interneuron are. Corresponding to a role in flight behavior, A4I1 makes direct connections with the motor neurons to the pleuroaxillary muscles of front and hind wings, as well as with an unidentified motor neuron to a muscle of the first abdominal segment (Figure 1D). The pleuroaxillary wing muscles are functional steering muscles since they control the angle of pronation and supination and, thus, adjust thrust and lift and function in all steering manoeuvres.

### Structural dynamics shape A4I1's receptive field?

In contrast to the number of and input from sensory receptors, the dendritic and axonal arbors of the A4I1-neuron do not dramatically change between first instars and adult locusts (Bucher and Pflüger, 2000). When the responses of the two A4I1-neurons to wind stimuli from different directions are recorded extracellularly, only quantitative changes are observed between nymphal instars and adults. In general, these changes are characterized by an increasing separation of the two neurons' receptive fields, such that only in adult animals, when flight emerges as a new behavior, the full directional sensitivity is acquired (Bucher and Pflüger, 2000).

The A4I1-neuron is not the only interneuron which receives inputs from the prothoracic wind hairs. An electrophysiological search in the prothoracic ganglion revealed additional interneurons, some with their somata within the prothoracic ganglion (Münch, 2006). Details of their connectivity and function remain as yet enigmatic, however.

### How does this hair-to-interneuron system function in (restrained) flight?

It is suggestive to speculate that the hair receptors of A4I1's complex receptive field monitor parameters of the air flow around the head and the frontal part of the thorax in a flying locust. Examining the air flow around a locust head with removed front legs shows that it is more or less laminar until the mesothoracic segment (Pflüger and Tautz, 1982) and that proepisternal hairs are deflected in air flow direction to a maintained position as long as the flow persists. Nothing is known about the proepisternal receptors, but if the front legs were fixed in the characteristic flight posture the air flow became turbulent, suggesting that this will also happen to the air flowing around the proepisternum.

In keeping with a role in flight behavior, output connections onto flight steering muscles suggest a role in course control. It would appear necessary to examine such hypotheses by, first, visualizing the air flow around the locust head and thorax in (tethered) flight and, second, observing possible responses to selective stimulation of the respective hair receptors in the A4I1 interneuron.

To approach the second aspect, we recorded intracellularly from the A4I1-soma and extracellularly from one pleuroaxillary muscle in a dissected locust flying upside down in front of a wind tunnel. Head wind speed was ~2 m/s, and during fictive flight small air puffs were delivered from a cut microelectrode at ~10-fold weaker wind speeds (20 cm/s). The opening of this microelectrode was placed opposite to the proepisternum and the space that is formed by the head and the first thoracic segment with the probasisternal hairs pointing into this space (indicated in Figure 3B). As shown in Figure 3A, the A4I1-interneuron with its axon ipsilateral (and soma contralateral) to the pipette is rhythmically excited already by the animals' own wing beat, even without any external wind stimulus (0–4 spikes per wingbeat cycle, 1.75 on average). The recorded activity represents spikes that passively invade the soma (see above) and are superimposed on depolarizations that reach the soma from the neurites. That is, the size relationships of spikes and subthreshold depolarizations are distorted. Nonetheless, A4I1 activity pattern is clearly discernible. An air puff from the pipette causes a complex response, an initial inhibition followed by a pronounced burst (asterisk). With a head wind of 2 m/s the A4I1 neuron is excited much more strongly than during stationary flight in resting air (Figure 3C) (1–5 spikes per wingbeat cycle, 2.61 on average). Nonetheless, a weak turbulent air puff is clearly reflected by a burst of spikes in the recording (asterisk). No inhibition is discernible and the excitatory response occurs much earlier than in the situation without head wind. Detailed interpretation of these observations is impossible at present since the (aerodynamic) mode of stimulation of the hair sensilla is not clear, and neither is the change in the air puff stimulus brought about by the head wind. It would appear possible that with head wind present the air puff is deflected and becomes more turbulent, thus stimulating different sets of hair receptors at different strengths, which may have caused the differences in the response characteristics.

In summary, we conclude that the A4I1 hair-to-interneuron system probably monitors weak turbulences around the anterior locust body during flight. In line with this interpretation, wind alone without flight motor activity already excites A4I1 above threshold (not shown). The characteristic flight posture of the front legs may further allow the animal to direct air flow into the afore-mentioned space and thus influence or modulate the wind stimulus reaching the probasisternal, proepisternal, and pronotal hairs. Again, further study is essential here to assess the validity of these ideas. With modern laser Doppler techniques such experiments appear actually quite feasible despite difficult access to some of the hair sensilla.

### Keeping A4I1 in a suitable working range

The mechanosensory-to-flight motor pathway from filiform hairs to wing steering muscles via the A4I1 interneuron makes sense in a flight steering context, as does the response of the A4I1 interneuron to air puffs just presented. However, the enormous sensitivity of the filiform hair-to-interneuron connection remains intriguing. Mechanisms must exist to prevent this system from working at or close to saturation.

A few candidate mechanisms exist that may prevent the hair-to-interneuron system from reaching saturation. Among them is presynaptic gain control, described for sensory afferents from chordotonal organs (Burrows and Matheson, 1994) in walking (Wolf and Burrows, 1995), and in stridulation (Poulet, 2005; Poulet and Hedwig, 2006). Although electrophysiological study of possible presynaptic inhibition is still missing, GABAergic mechanisms are clearly in place to limit A4I1-firing (Gauglitz and Pflüger, 2001). In addition, the prothoracic neuropile is densely labeled by NO-synthase-immunoreactive profiles (Münch et al., 2010) in areas where synaptic interactions between the filiform hair receptors and the A4I1-neuron occur. And NO has been shown to effect a general decrease in the A4I1 response to a wind-puff (Münch et al., 2010).

## Conclusions

Not just in holometabolous insects but in hemimetabolous insects as well, sensory and motor neurons may exhibit remarkable structural and functional dynamics, dependent on the respective developmental context. In addition to hormonal regulation, which provides a developmentally programmed time frame, activity-dependent mechanisms adjust sensory receptors to individual characters. This is evident when sensory receptors are ablated and synaptic rearrangement including structural dynamics occurs, even in adult insects. For example, interneurons may connect to sensory receptors they would never receive input from under normal conditions (Murphey, 1986; Brodfuehrer and Hoy, 1988; Kanou et al., 2004). The locust filiform hair-to-interneuron system involving A4I1 is suitable for such studies, particularly with regard to its well-known output connections, by comparison to other systems.

### Conflict of interest statement

The authors declare that the research was conducted in the absence of any commercial or financial relationships that could be construed as a potential conflict of interest.
